# Haemochromatosis patients' research priorities: Towards an improved quality of life

**DOI:** 10.1111/hex.13830

**Published:** 2023-07-28

**Authors:** Lídia Romero‐Cortadellas, Veronica Venturi, Juan Carlos Martín‐Sánchez, Ketil Toska, Dianne Prince, Barbara Butzeck, Graça Porto, Nils Thorm Milman, HI/EFAPH Survey Committee, Mayka Sánchez

**Affiliations:** ^1^ Department of Basic Sciences, Iron metabolism: Regulation and Diseases Universitat Internacional de Catalunya (UIC) Sant Cugat del Vallès Barcelona Spain; ^2^ Group of Evaluation of Health Determinants and Health Policies, Department of Basic Sciences Universitat Internacional de Catalunya Sant Cugat del Vallès Spain; ^3^ Norwegian Haemochromatosis Association Bergen Norway; ^4^ Haemochromatosis Australia Meridan Plains Queensland Australia; ^5^ Hämochromatose‐Vereinigung Deutschland e.V. HVD European Federation of Associations of Patients with Haemochromatosis (EFAPH) Hattingen Germany; ^6^ i3S—Instituto de Investigação e Inovação em Saúde Universidade do Porto Porto Portugal; ^7^ ICBAS—Instituto de Ciências Biomédicas Abel Salazar. Universidade do Porto Porto Portugal; ^8^ Danish Hemochromatosis Association Copenhagen Denmark; ^9^ EFAPH—European Federation of Associations of Patients with Haemochromatosis Croissy‐sur‐Seine France; ^10^ BloodGenetics S.L. Diagnostics in Inherited Blood Diseases Esplugues de Llobregat Spain

**Keywords:** chronic disease, haemochromatosis, iron overload, patients' needs, quality of life (QoL), survey

## Abstract

**Background:**

Chronic diseases are associated with a range of functional and psychosocial consequences that can adversely affect patients' quality of life (QoL). Haemochromatosis (HC) is a genetically heterogeneous disorder characterized by chronic iron overload that can ultimately lead to multiple organ dysfunction. Clinical diagnosis remains challenging due to the nonspecificity of symptoms and a lack of confirmatory genotyping in a substantial proportion of patients. Illness perception among HC patients has not been extensively investigated, lacking relevant information on how to improve their QoL.

**Methods:**

We present the results of the first worldwide survey conducted in nearly 1500 HC respondents, in which we collected essential demographic information and identified the aspects that concern HC patients the most.

**Results:**

Out of all the participants, 45.3% (*n* = 676) voiced their concern about physical and psychological consequences such as HC‐related arthropathies, which can ultimately affect their social functioning. A similar proportion of patients (*n* = 635, 42.5%) also consider that better‐informed doctors are key for improved HC disease management. Taking a patient‐centred approach, we expose differences in patients' disease perspective by social and economic influences.

**Conclusions:**

We identify potential targets to improve patients' health‐related QoL and reflect on strategic measures to foster gender equity in access to health resources. Finally, we make a call for a highly coordinated effort across a range of public policy areas to empower participants in the HC research process and design.

**Patient or Public Contribution:**

Nearly 1500 patients with hereditary HC responded to an anonymized online survey in which research and clinical priorities were addressed regarding this chronic and rare disease.

## 1 INTRODUCTION

Patient‐centred care is an evolving concept, initially conceived as the understanding of patients as unique individuals.^1^ In agreement with Ekman et al,^2^ a better term for this idea is ‘person‐centred care’, as it refrains from reducing the person to just their symptoms and/or disease. At the global level, the World Health Organization (WHO) has developed policy frameworks for person‐centred health care.^3^ Traditionally, researchers and funding programs have determined which research topics were worth pursuing. Nowadays, however, patients' involvement is no longer limited to issues related to clinical practice; instead, it has opened up to collaborations that improve the way research procedures are prioritized, communicated and carried out.^4^, ^5^ Patients report feeling more confident and satisfied when contributing to knowledge and the process has been found to be educational for all parties involved.^5^ Nonetheless, life‐threatening illnesses have been given preferential attention with the goal of diagnosing, treating and curing the patient. This focus often ignores the vast number of adults living with chronic and/or disabling genetic conditions that persist and may progressively worsen over a lifetime.^6^

Haemochromatosis (HC) is a genetically heterogeneous disorder and one of the most common genetic diseases in European‐descendant populations.^7^, ^8^ As a chronic iron overload condition, HC is characterized by excessive absorption of iron due to hepcidin deficiency.^7^ Over the last decades, HC diagnosis has improved with the discovery of several genes involved in its aetiology; be that as it may, the management of the disease has remained unchanged and phlebotomy prevails as the standard treatment for HC patients.^7^ The liver is typically the most affected organ due to iron accumulation, but early diagnosis and an adequate treatment have reduced the mortality associated with hepatic alterations. At present, chronic fatigue is the most common early symptom encountered in the disease course, while bone and joint complications, for example, osteoporosis and arthropathy, have also been consistently described in HC patients by the fifth or sixth decade of life.^7^ Most of these conditions seem not to be improving with treatment and the mechanisms leading to impairment of bone metabolism in HC remain poorly understood.^9^

Despite the progress made in HC management, its clinical diagnosis remains challenging due to nonspecific early symptoms and the incomplete penetrance of a highly frequent HC‐causing genetic variant; hence, it heavily relies on physicians' knowledge of HC and informative biochemical tests.^10^ Predictably, delayed diagnosis can be associated with major complications such as hepatocellular carcinoma.^11^ This has prompted to implement recommendations for improved early case detection, which translated into timely treatment before symptoms onset.^7^ That said, the HC diagnostic process continues to be conducted without confirmatory genotyping in a substantial proportion of patients while results are frequently misinterpreted. This ultimately leads to misdiagnoses and inappropriate therapeutic plans, two widespread clinical problems in HC management.^12^

Illness perception in HC patients has not been extensively investigated,^13^ lacking relevant information on matters perceived as important to improve their quality of life (QoL). We address this matter in the present study wherein HC patients from 23 countries were invited to take part in an anonymous online survey to determine which issues concern HC patients the most and should therefore be prioritized in the research field and/or at the clinical level.

## 2 MATERIALS AND METHODS

### 2.1 The form

A first Google form was proposed and later reviewed and approved by the European Federation of Associations of Patients with Haemochromatosis/Haemochromatosis International (EFAPH/HI) joint scientific committee. The questionnaire design and the candidate areas of enquiry were agreed upon by committee members who contribute to the content write‐up in their area of expertise. The form was then translated from English into several languages including Danish, Dutch, Finnish, French, German, Hungarian, Icelandic, Italian, Norwegian, Polish, Portuguese, Spanish and Swedish, with the help of local HC associations board members. Survey links were made available to all HC associations, which were in charge of disseminating it among HC patients via both E‐mail and social networks. HC patient associations from the following countries participated in the study: Australia, Austria, Belgium, Brazil, Canada, Denmark, France, Germany, Hungary, Ireland, Italy, New Zealand, Norway, Portugal, Spain, Sweden, the Netherlands, the United Kingdom and the United States of America. Moreover, the survey was sent to patients in Argentina, Finland, Iceland and Poland, where formal HCs associations are not yet officially constituted. Patients had access to the forms between December 22, 2020 and May 5, 2021.

### 2.2 Data collection

To characterize the sample, demographic data were collected from every individual including sex assigned at birth, age group (in 10‐year intervals), nationality (continent), educational level, occupation and affiliation length to an HC association. Participants were asked to select three research priorities in the field of HC out of the following options:

1. Conduct research into arthritis and joint problems.
2. Conduct research into impotence and hormonal problems.
3. Conduct research into extreme and chronic fatigue.
4. Conduct research into psychological or cognitive difficulties (mood swings, ‘brain fog’, etc.).
5. Identify best practices for diagnosis and treatment in all countries and regions.
6. Investigate new or alternative treatments for HC.
7. Investigate methods to improve phlebotomies.
8. Investigate new genes that can be involved in HC.
9. Promote equal access to genetic diagnosis in all suspected cases.
10. Harmonize the use of blood from HC patients in all countries.
11. Research the epidemiological aspects of HC, and conduct more studies on its prevalence (proportion of population found to be affected) and geographical distribution.
12. Implement transferrin saturation tests in the initial and maintenance phase.
13. Promote health literacy and knowledge about HC among patients and the general population.
14. Promote knowledge about HC among medical doctors.
15. Other (not listed above).

Respondents could contribute with their opinion by selecting the last option ‘*Other (not listed above)*’. All free written answers were recoded into 13 categories according to their content: symptomatology and comorbidities, diagnosis, general population HC awareness, treatment, knowledge among physicians, blood donation, diet, genes, coronavirus disease 2019 (COVID‐19), arthritis, disease epidemiology, access to medical specialists and agreement with all the options.

### 2.3 Analysis

Data obtained in the study were analysed with Microsoft Excel 2016 and jamovi 2.3.7, and graphed with GraphPad Prism 9. A *χ* ^2^ test was used to compare the distribution of answers among demographic categories. Groups with <10 participants (Table 1) were not included in the analysis for that particular category, namely: ‘Other’ (sex assigned at birth), ‘17 or younger’ (age), Africa and Asia (continent); and ‘Student’ (employment). Furthermore, Option 15 ‘*Other (not listed above)*’ was excluded from the analysis as free written answers were considered separately.

**Table 1:** Demographic characteristics of the participants included in the study.

	Number	Percentage
Sex assigned at birth		
Female	832	55.7
Male	659	44.2
Other	2	0.1
Age		
17 or younger	2	0.1
18–29	26	1.7
30–39	91	6.1
40–49	259	17.4
50–59	415	27.8
60–69	427	28.6
70–79	236	15.8
80 or older	37	2.5
Continent		
Africa	3	0.2
Asia	2	0.1
Australia/Oceania	189	12.7
Europe	954	63.9
North America	265	17.7
South America	80	5.4
Education		
Did not complete high school	139	9.3
High school certificate	472	31.6
Unfinished university studies	165	11.1
Bachelor's degree	422	28.3
Postgraduate	295	19.8
Occupation		
Employed	789	53.4
Unemployed	51	3.4
Retired	521	34.9
Unable to work because of disability	113	7.6
Student	10	0.7
Haemochromatosis association membership		
Never	182	12.2
Less than a year	335	22.4
1–5 years	480	32.2
5–10 years	236	15.8
More than 10 years	260	17.4

### 2.4 Ethics approval and consent

The protocol was approved by the Ethics Committee of the Hospital General de Catalunya on October 24, 2018 (2018/74‐HEM_UIC‐HUGC).

## 3 RESULTS

A total of 1772 HC patients answered the questionnaire. Out of these, 279 respondents (15.7%) were excluded because they had listed more than three answers in the last question; this was a stated requisite in the questionnaire but for technical issues it was not possible to limit the number of responses. The answers of the remaining 1493 participants were considered for further analysis. About 55.7% of these were females, whereas males represented 44.1%, with about 75% of the population aged >50 years, consistent with HC age of onset. More than half of the participants were employed (53.4%) and a significant number of them were already retired (34.9%). Of note, 12.2% of the participants had never been affiliated with any HC association. Other demographic characteristics of the participants are described in Table 1.

**Figure 1:**
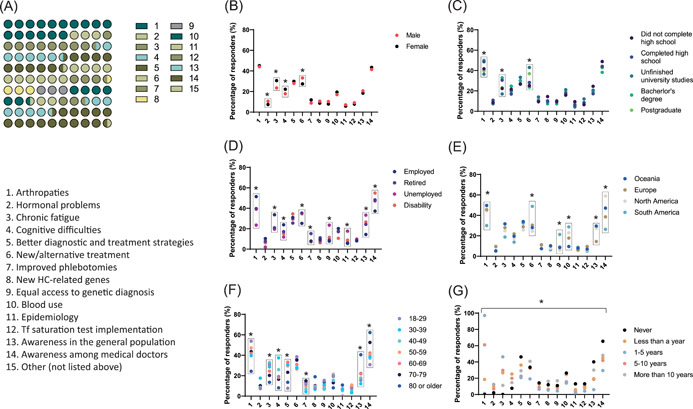
Analysis of patients' research priorities on haemochromatosis (HC). (A) Global percentage distribution of observations, ordered by the answer number. Each dot corresponds to 1%. Values in the plot are detailed in Table 3, third column. (B–G) Distribution of selected options grouped by demographic variables: sex assigned at birth (B), educational level (C), employment situation (D), continent (E), age (F) and time belonging to an HC association (G). A *χ* ^2^ test was performed to analyse differences between the groups (see Section 2). All groups in (G) showed statistical differences. Analysis results are detailed in Supporting Information: Tables S1–6. Tf, transferrin. *p < .005.

When analysing patients' preferences in HC research, they selected *Conduct research into arthritis and joint problems* as the top priority in the HC field (45.3%), closely followed by *Promote knowledge about* HC among medical doctors, with 42.5% (Table 3 and Figure 1A). The third most voted choice, *Investigate new or alternative treatments for* HC, was indicated by 30% of participants. On the other hand, the least preferred answer options were *Conduct research into impotence and hormonal problems* (8.8%), *Implement transferrin saturation tests in initial and maintenance phase* (8.4%) and *Research the epidemiological aspects of HC* (6.4%).

Gender‐specific data analysis revealed that significant differences existed between the two sexes (Figure 1B and Supporting Information: Table S1): women prioritized conducting research in extreme, chronic fatigue and psychological or cognitive difficulties, while men were more concerned about impotence, hormonal problems and new treatments for HC. When grouping participants by their educational background, no foreseeable, relevant differences were found between the parties, suggesting that academic achievement level exerts limited influence over patients' disease perspective (Figure 1C and Supporting Information: Table S2).

The answer distribution across different employment statuses showed that the workforce significantly values research on topics that may adversely affect their fitness for work, including research on arthropathies, chronic fatigue. and cognitive difficulties (Figure 1D and Supporting Information: Table S3). Besides, HC patients who are unable to work due to their health condition were the most preoccupied with their physicians being adequately knowledgeable about HC. Respondents living in low‐income countries (i.e., South America)^14^ claimed for more research on new HC treatments, equal access to genetic diagnostics and the harmonization of blood donation after phlebotomies, whereas they placed arthropathy research at the bottom of the HC research agenda, suggesting an economic bias (Figure 1E and Supporting Information: Table S4).

An increasing percentage of participants across age groups considered that HC is still unsatisfactorily understood both by the general population and at the clinical level (Figure 1F and Supporting Information: Table S5). Interestingly, older age groups (>70 years) showed no major preoccupation with signs and symptoms that typically affect the elderly including arthritis, fatigue or cognitive impairment.^15^ The data analysed would indicate that preferences in HC research change throughout life, possibly influenced by the disease course and other associated physiological and psychological changes such as internal adaptive coping strategies and positive interpretations of disease.^15^ Unexpectedly, the answer distribution had a high dispersion for all options when patients were grouped according to their length of affiliation with an HC association (Figure 1G and Supporting Information: Table S6)

Finally, 340 participants (22.8%) included a free written answer by selecting the option ‘*Other (not listed above)*’. A brief summary of these data is available in Table 2. Most of the comments remarked on issues already included in the survey's answer options. However, it is noteworthy that 17.4% of these individuals referred to the need to impulse research on HC implications in other diseases such as pain disorders, gut problems or early onset of neurological and cognitive affections. Furthermore, 2.9% stated that improved dietary guidelines should be provided to HC patients to minimize iron intake. Interestingly, some participants (2.7%) were concerned about the possible effects of severe acute respiratory syndrome coronavirus 2 (SARS‐CoV‐2) infection and/or of the COVID‐19 vaccines on HC patients.

**Table 2:** Summary of recorded written answers.

Group	Number	Percentage
Symptomatology and comorbidities	59	17.4
Diagnosis	55	16.2
General population haemochromatosis awareness	50	14.7
Treatment	41	12.1
Knowledge among physicians	40	11.8
Blood donation	18	5.3
Diet	10	2.9
Genes	10	2.9
COVID‐19	9	2.7
Arthritis	4	1.2
Disease epidemiology	4	1.2
Access to medical specialists	2	0.6
Agreement with all the options	38	11.2
Total	340	100

Abbreviation: COVID‐19, coronavirus disease 2019.

**Table 3:** Number and percentage of participants that selected each research priority.

	Number of respondents	Percentage from total individuals	Percentage from total answers
1. Arthropathies	676	45.3	15.8
2. Hormonal problems	132	8.8	3.1
3. Chronic fatigue	410	27.5	9.6
4. Cognitive difficulties	304	20.4	7.1
5. Better diagnostic and treatment strategies	434	29.0	10.2
6. New/alternative treatment/s	448	30.0	10.4
7. Improved phlebotomies	157	10.5	3.7
8. New HC‐related genes	143	9.6	3.3
9. Equal access to genetic diagnosis	135	9.0	3.1
10. Blood donation after phlebotomy	273	18.3	6.4
11. HC epidemiology	96	6.4	2.2
12. Transferrin saturation test implementation	125	8.4	2.9
13. HC awareness in the general population	289	19.4	6.7
14. HC awareness among medical doctors	635	42.5	14.8
15. Other (not listed above)	32	2.1	0.7
Total	4289	287.3	100

*Note*: The percentage of individuals (out of 1493) who chose options 1–15 is displayed in the second column, while the percentage that each option represents (out of 4289 total answers) corresponds to the third column. As some individuals (*n* = 190) did choose one or two research priorities out of the three possible, the total percentage of individuals choosing each alternative does not reach 300.

Abbreviation: HC, haemochromatosis.

## 4 DISCUSSION

In this study, we present the first worldwide survey intended to identify HC patients' needs, their perspectives on the disease and future research priorities. The results of the questionnaire reveal that HC patients are largely concerned about arthritis and other joint problems derived from their condition; this finding is congruent with arthropathy being an early symptom of HC, which can severely affect up to 81% of subjects.^9^, ^16^, ^17^ In HC patients aged younger than 60 years, primary joint replacement surgeries are needed earlier in life compared with the general population; both arthropathies associated with blood disorders and the correlated orthopaedic surgery procedures can be a cause of morbidity and disability, negatively affecting patients' QoL.^18^, ^19^ It remains unclear how this condition develops in these patients and whether it could be prevented by an earlier intervention with antirheumatic drugs. Moreover, conventional phlebotomy treatment in HC patients seems to have minimal benefits in preventing or ameliorating arthropathies.^7^ It is therefore reasonable that patients, particularly the working‐age population, claim for more research in this area. Committed to raising awareness about iron metabolism and arthropathy, HI and EFAPH have been taking action to promote and support the creation of the HC Arthropathy Research Initiative.^20^

Of note, female participants demanded more investigation on extreme and chronic fatigue (Figure 1). This could denote that, even though this is one of the most common disease symptoms, it may be more prevalent among women and should be addressed accordingly to dismiss gender‐based health inequalities. In younger individuals, HC symptoms could overlap with the hormonal changes they may be experiencing, while later in life they could be mistaken for disturbances proper of the menopausal transition.^21^ Contrarily, HC‐associated hormonal problems seem to mainly concern the male counterpart. This does not come entirely unexpected given that loss of libido and impotence can present as early, nonspecific symptoms in up to 40% of the homozygous male patients.^22^ In view of the qualitative and quantitative studies on sexual dysfunctions reporting on a diminished perception of both masculinity and self‐worth,^23^ our survey result warrants more research on HC‐related sexual health issues.

Participants also pointed to the unmet need to foster knowledge of HC among medical doctors, as well as implementing best practices for HC diagnosis; this leaves us to ponder whether alleviating such a degree of uncertainty and/or partial lack of confidence towards health professionals could somehow improve patients' disease outcome. Despite the dedicated work of patient associations on this issue, our study consistently suggested that the outcome of this effort is limited and especially worrisome for disabled patients, which could imply unsatisfactory past experiences.^13^ Furthermore, some individuals lamented a lack of nutritional recommendations by their physicians and called for more informative dietary guidelines for HC patients. Educational initiatives targeting physicians would plausibly improve both HC underdiagnosis and counselling support for this disease.

Another aspect to take into consideration is the patients' demand for new HC treatments to be investigated. Currently, HC therapy is mostly based on an initial phase of fast iron depletion by repeated phlebotomies, which requires regular visits to the hospital and, in turn, limits patients' autonomy; this is followed by a less demanding maintenance phase. Iron chelation therapy remains a valid alternative to phlebotomies in some patients and improved formulations of chelating agents are being investigated to minimize their side effects.^24^, ^25^ Other treatment strategies include erythrocytapheresis, a procedure by which erythrocytes are separated from whole blood; or the prescription of proton pump inhibitors, particularly valuable in patients that cannot undergo phlebotomies.^7^

HC is associated with significant healthcare costs and utilization driven by outpatient visits, pharmaceutical claims and comorbidities.^26^, ^27^ Reasonably, patients living in countries with limited economic resources demanded improved HC treatment and facilitated access to genetic diagnostic, a primary tool for quality of care and positive patient outcomes. Screening high‐risk individuals, that is, first‐degree relatives with a history of HC, has been deemed strategic to increase the rates of early detection in the precirrhotic stage,^28^, ^29^ thereby lessening HC clinical and economic burdens. Respondents from low‐income countries also claimed for the harmonization of blood use after phlebotomies, reflecting a lack of common policies concerning the use of blood and haemoderatives obtained from HC patients. Regulations are highly variable among different countries: while some allow blood usage if patients meet general donation requirements, others discard it. Studies report that the quality of red cell concentrates and platelets from HC individuals are comparable to those of routine donors, although in vitro experiments suggest that units from iron‐overloaded patients may be more susceptible to bacterial growth.^30^ Extending the blood donor eligibility criteria to asymptomatic HC individuals would presumably improve their emotional well‐being by fulfilling a sense of purpose, as well as serve society by expanding the blood donor pool.^30^

Responding to uncertainty in health care associated with COVID‐19, a proportion of patients expressed their preoccupation with the repercussions of the pandemic on their health status. Mounting evidence points to the detrimental outcomes of viral infections, including SARS‐CoV‐2, on iron‐overloaded individuals.^31^, ^32^ It seems, therefore, reasonable that underlying metabolic disturbances could be a risk factor for the development of severe COVID‐19 in HC patients. Adding to this, the pandemic outbreak posed significant barriers to diagnosis, treatment and follow‐up of chronic diseases as demanding as HC in terms of hospital outpatient treatment.^33^

On a different scale of importance, participants considered that epidemiological aspects of HC—the least voted option—are not an element of concern. In fact, due to its high prevalence in certain populations, HC distribution has been extensively studied, particularly since the discovery of the *HFE* gene in 1996 and of the so‐called ‘Celtic mutation’.^34^, ^35^, ^36^ However, caution should be exercised in drafting bold conclusions: the term ‘epidemiological’, included in the item stem, may have been unfamiliar to some respondents, affecting the validity of the conclusions. Participants also took little interest in the implementation of transferrin saturation tests, most probably due to limited knowledge of their utility.

The outcome of this patient‐centred investigation uncovers previously undiscussed patients' needs and satisfaction targets and offers valuable points of reasoning for more rigorous research and design. Gender‐inclusive studies should address concerns raised by women of all age groups: it would be informative, for instance, to corroborate whether chronic fatigue can be classified as a strictly correlated HC symptom or may have been overreported in some patient age groups. When focusing on medically vulnerable populations such as the elderly, our findings align with adaptation dynamics experienced in older patients coping with lifelong unremitting symptoms such as reappraisal attitude and social–cognitive processes of life reflection.^15^ In view of this, it may be relevant to elaborate on these adaptive coping strategies so that health professionals could explore them in a clinical setting. The impact of chronic medical conditions on both productivity loss and work impairment has been extensively documented; this also emerges as a source of concern among our respondents who demand a research effort to improve aspects that hinder their daily performance, including joint problems, chronic fatigue and recurrent appointments to health centres.^37^, ^38^, ^39^, ^40^ As HC might result in functional and cognitive limitations that impair employees' ability to fully participate in the workforce, efforts should be made to enable sustainable employment and avert social exclusion in the labour market. Patients' demand for more genetic testing fuels the debate on the eligibility for diagnostic of this sort; as for HC screening programs, a consensus on the health economic value of a population‐based approach is yet to be reached.^29^, ^41^, ^42^ Irrespective of the limitations cited in health economic evaluations,^43^, ^44^ the declining cost of genetic testing may shift the cost‐effectiveness outcome and the way we assess healthcare options in the near future. When considering socioeconomic status (SES), our research concludes that education is not a driving parameter when contributing to diseases' perception, while income and occupation are. Better targeted SES examinations are likely to be needed to expose inequities in access to health resources, for example, genetic testing and treatment facilities. Finally, as the survey was distributed worldwide, a degree of cultural response bias cannot be discarded as the combined impact of cultural respondent tendency and expectation is difficult to discern.

A committed partnership between all parties appears necessary to develop effective policies and implement guidelines that could globally improve HC medical management. More ambitiously, we should aspire to a global framework, ideally not limited to a disease‐specific approach, to implement strategic actions for a broad range of chronic conditions.

## AUTHOR CONTRIBUTIONS

*Study concept and survey design*: Mayka Sánchez, Dianne Prince, Ketil Toska, Barbara Butzeck, Graça Porto, Nils Thorm Milman and HI/EFAPH. *Acquisition of data and analysis*: Lídia Romero‐Cortadellas and Veronica Venturi. *Drafting of the manuscript*: Veronica Venturi, Lídia Romero‐Cortadellas and Mayka Sánchez. *Statistical analysis*: Lídia Romero‐Cortadellas, Veronica Venturi and Juan Carlos Martín‐Sánchez. *Obtaining funding*: Mayka Sánchez. All authors have read and agreed to the published version of the manuscript.

## CONFLICT OF INTEREST STATEMENT

The authors declare no conflict of interest.

## ETHICS STATEMENT

The protocol was approved by the Ethics Committee of the Hospital General de Catalunya on October 24, 2018 (2018/74‐HEM_UIC‐HUGC).

## Supporting information

Supporting information.

## Data Availability

The data that support the findings of this study are available from the corresponding author upon reasonable request.
