# Supplementary material for: Haemochromatosis patients' research priorities: Towards an improved quality of life
Source: Health Expect. 2023 Jul 28;26(6):2293–301. doi: 10.1111/hex.13830 (PMC10632644; doi:10.1111/hex.13830)
Supplement: Supplementary file 1 — Supporting information. [file HEX-26--s001.docx]

**Supplementary material for**

**Hemochromatosis patients’ research priorities: towards an improved quality of life**

Lídia Romero-Cortadellas^1¶^, Veronica Venturi^1¶^, Juan Carlos Martín-Sánchez^2^, Ketil Toska^3^, Dianne Prince^4^, Barbara Butzeck^5^, Graça Porto^6^, Nils Thorm Milman^7^, HI/EFAPH Survey Committee^8^, Mayka Sánchez^1,9**^.

^¶^ Both authors contributed equally to this paper

** Corresponding author

1 Department of Basic Sciences, Iron metabolism: Regulation and Diseases. Universitat Internacional de Catalunya (UIC), Sant Cugat del Vallès, Spain. [lromerocor@uic.es](mailto:lromerocor@uic.es) (L.R-C.), [vventuri@uic.es](mailto:vventuri@uic.es) (V.V.), [msanchezfe@uic.es](mailto:msanchezfe@uic.es) (M.S.)

2 Group of Evaluation of Health Determinants and Health Policies, Department of Basic Sciences, Universitat Internacional de Catalunya, Sant Cugat del Vallès, Spain. [jcmartin@uic.es](mailto:jcmartin@uic.es) (JC. M-S.)

3 Norwegian Haemochromatosis Association, Bergen, Norway. [ketil.toska@mintra.com](mailto:ketil.toska@mintra.com) (K.T.)

4 Haemochromatosis Australia, Meridan Plains, Australia. [dianne.prince@haemochromatosis-international.org](mailto:dianne.prince@haemochromatosis-international.org) (D.P.)

5 Hämochromatose-Vereinigung Deutschland e.V. HVD, European Federation of Associations of Patients with Haemochromatosis EFAPH, Hattingen, Germany. [barbara.butzeck@gmx.de](mailto:barbara.butzeck@gmx.de) (B.B.)

6 i3S—Instituto de Investigação e Inovação em Saúde, Universidade do Porto, CHUPorto-Centro Hospitalar Universitário do Porto; ICBAS-Instituto de Ciências Biomédicas Abel Salazar, Universidade do Porto, Portugal. [gporto@ibmc.up.pt](mailto:gporto@ibmc.up.pt) (G.P.)

7 Danish Hemochromatosis Association, Copenhagen, Denmark. [nils.milman@outlook.com](mailto:nils.milman@outlook.com) (N.T.M.)

8 EFAPH-European Federation of Associations of Patients with Haemochromatosis, Croissy-sur-Seine, France. [info@efaph.eu](mailto:info@efaph.eu) (HI/EFAPH SC)

9 BloodGenetics S.L. Diagnostics in Inherited Blood Diseases. Esplugues de Llobregat, Spain. [msanchezfe@uic.es](mailto:msanchezfe@uic.es) (M.S.)

**Correspondence**

Dr. Mayka Sánchez. Universitat Internacional de Catalunya (UIC), Department of Basic Sciences, Iron metabolism: Regulation and Diseases. Sant Cugat del Vallès, Barcelona, Spain. Telephone: +34 935 042 000 (ext 5263). Email: [msanchezfe@uic.es](mailto:msanchez@uic.es)

**Table S1. Answer distribution analysis compared by gender**. The number and percentage of individuals within the same group that chose each option is given. The gender option “Other” has been excluded as n<10 (see Materials and Methods).

|  | **Male** | **Female** | **X^2^ p-value** |
| --- | --- | --- | --- |
| 1. Arthropathies | 301 (45.1%) | 375 (45.1%) | 0.82 |
| 2. Hormonal problems | 70 (10.6%) | 62 (7.5%) | **0.03** |
| 3. Chronic fatigue | 155 (23.5%) | 255 (30.6%) | **0.02** |
| 4. Cognitive difficulties | 118 (17.9%) | 185 (22.2%) | **0.04** |
| 5. Better diagnostic and treatment strategies | 184 (27.9%) | 249 (29.9%) | 0.4 |
| 6. New/alternative treatment/s | 219 (33.2%) | 228 (27.4%) | **0.02** |
| 7. Improved phlebotomies | 58 (8.8%) | 99 (11.9%) | 0.05 |
| 8. New HC-related genes | 70 (10.6%) | 72 (8.7%) | 0.2 |
| 9. Equal access to genetic diagnosis | 68 (10.3%) | 66 (7.9%) | 0.11 |
| 10. Blood donation after phlebotomy | 112 (17.0%) | 161 (19.4%) | 0.24 |
| 11. HC epidemiology | 47 (7.1%) | 49 (5.9%) | 0.33 |
| 12. Transferrin saturation test implementation | 60 (9.1%) | 65 (7.8%) | 0.37 |
| 13. HC awareness in the general population | 136 (20.6%) | 153 (18.4%) | 0.28 |
| 14. HC awareness among medical doctors | 273 (41.4%) | 361 (43.4%) | 0.45 |
| 15. Other (not listed above) | 13 (2.0%) | 19 (2.3%) | 0.68 |

**Table S2. Answer distribution analysis compared by the educational background.** The number and percentage of individuals within the same group that chose each option is given. Significant differences in the Chi-square test are highlighted in bold.

|  |  | **Did not complete high school** | **Completed high school** | **Unfinished university studies** | **Bachelor's’ degree** | **Postgraduate** | **X^2^ p-value** |
| --- | --- | --- | --- | --- | --- | --- | --- |
| 1. Arthropathies | | 58 (41.7%) | 230 (48.7%) | 60 (36.4%) | 211 (50.0%) | 117 (39.7%) | **0.003** |
| 2. Hormonal problems | | 12 (8.6%) | 51 (10.8%) | 12 (7.3%) | 33 (7.8%) | 24 (8.1%) | 0.48 |
| 3. Chronic fatigue | | 31 (22.3%) | 144 (30.5%) | 28 (17%) | 139 (32.9%) | 68 (23.1%) | **< .001** |
| 1. Cognitive difficulties | | 30 (21.6%) | 80 (16.9%) | 33 (20.0%) | 104 (24.6%) | 57 (19.3%) | 0.08 |
| 1. Better diagnostic and treatment strategies | | 37 (26.6%) | 127 (26.9) | 55 (33.3%) | 127 (30.1%) | 88 (29.8%) | 0.52 |
| 1. New/alternative treatment/s | | 35 (25.2%) | 133 (28.2%) | 71 (43.0%) | 100 (23.7%) | 109 (36.9%) | **< .001** |
| 1. Improved phlebotomies | | 13 (9.4%) | 47 (10.0%) | 23 (13.9%) | 40 (9.5%) | 34 (11.5%) | 0.52 |
| 1. New HC-related genes | | 20 (14.4%) | 48 (10.2%) | 15 (9.1%) | 31 (7.3%) | 29 (9.8%) | 0.17 |
| 1. Equal access to genetic diagnosis | | 13 (9.4%) | 45 (9.5%) | 17 (10.3%) | 31 (7.3%) | 29 (9.8%) | 0.70 |
| 1. Blood donation after phlebotomy | | 22 (15.8%) | 84 (17.8%) | 35 (21.2%) | 74 (17.5%) | 58 (19.7%) | 0.71 |
| 1. HC epidemiology | | 13 (9.4%) | 28 (5.9%) | 7 (4.2%) | 26 (6.2%) | 22 (7.5%) | 0.4 |
| 1. Transferrin saturation test implementation | | 9 (6.5%) | 40 (8.5%) | 20 (12.1%) | 36 (8.5%) | 20 (6.8%) | 0.32 |
| 1. HC awareness in the general population | | 34 (24.5%) | 81 (17.2%) | 34 (20.6%) | 84 (19.9%) | 56 (19.0%) | 0.4 |
| 1. HC awareness among medical doctors | | 68 (48.9%) | 208 (44.1%) | 72 (43.6%) | 161 (38.2%) | 126 (42.7%) | 0.2 |
| 1. Other (not listed above) | | 6 (4.3%) | 13 (2.8%) | 2 (1.2%) | 5 (1.2%) | 6 (2.0%) | 0.26 |

**Table S3. Answer distribution analysis compared by the employment situation.** The number and percentage of individuals within the same group that chose each option is given. The option “student” has been excluded as n<10 (see Materials and Methods). Significant differences in the Chi-square test are highlighted in bold.

|  | **Employed** | **Retired** | **Unemployed** | **Disability** | **X^2^ p-value** |
| --- | --- | --- | --- | --- | --- |
| 1. Arthropathies | 410 (51.4%) | 207 (39.7%) | 12 (23.5%) | 44 (38.9%) | **< .001** |
| 2. Hormonal problems | 83 (10.4%) | 40 (7.7%) | 1 (2.0%) | 7 (6.2%) | 0.06 |
| 3. Chronic fatigue | 268 (33.6%) | 109 (20.9%) | 10 (19.6%) | 22 (19.5%) | **< .001** |
| 4. Cognitive difficulties | 190 (23.8%) | 89 (17.1%) | 6 (11.8%) | 17 (15.0%) | **0.004** |
| 5. Better diagnostic and treatment strategies | 247 (31.0%) | 133 (25.5%) | 15 (29.4%) | 39 (34.5%) | 0.11 |
| 6. New/alternative treatment/s | 201 (25.2%) | 184 (35.3%) | 18 (35.3%) | 39 (34.5%) | **< .001** |
| 7. Improved phlebotomies | 63 (7.9%) | 79 (15.2%) | 4 (7.8%) | 8 (7.1%) | **< .001** |
| 8. New HC-related genes | 74 (9.3%) | 58 (11.1%) | 4 (7.8%) | 7 (6.2%) | 0.36 |
| 9. Equal access to genetic diagnosis | 62 (7.8%) | 47 (9.0%) | 12 (23.5%) | 13 (11.5%) | **0.001** |
| 10. Blood donation after phlebotomy | 161 (20.2%) | 89 (17.1%) | 9 (17.6%) | 12 (10.6%) | 0.08 |
| 11. HC epidemiology | 45 (5.6%) | 32 (6.1%) | 9 (17.6%) | 10 (8.8%) | **0.006** |
| 12. Transferrin saturation test implementation | 65 (8.1%) | 43 (8.3%) | 5 (9.8%) | 10 (8.8%) | 0.97 |
| 13. HC awareness in the general population | 114 (14.3%) | 126 (24.2%) | 17 (33.3%) | 30 (26.5%) | **< .001** |
| 14. HC awareness among medical doctors | 297 (37.2%) | 251 (48.2%) | 24 (47.1%) | 62 (54.9%) | **< .001** |
| 15. Other (not listed above) | 8 (1.0%) | 19 (3.6%) | 2 (3.9%) | 2 (1.8%) | **0.01** |

**Table S4.** **Answer distribution analysis compared by the continent of origin.** The number and percentage of individuals within the same group that chose each option is given. The options “Africa ” and “Asia'' have been excluded as n<10 (see Materials and Methods). Significant differences in the Chi-square test are highlighted in bold.

|  | **Europe** | **Oceania** | **North America** | **South America** | **X^2^ p-value** |
| --- | --- | --- | --- | --- | --- |
| 1. Arthropathies | 435 (45.6%) | 94 (49.7%) | 118 (44.5%) | 24 (30.0%) | **0.03** |
| 2. Hormonal problems | 92 (9.6%) | 10 (5.3%) | 24 (9.1%) | 4 (5.0%) | 0.16 |
| 3. Chronic fatigue | 267 (28.0%) | 60 (31.7%) | 66 (24.9%) | 15 (18.8%) | 0.12 |
| 4. Cognitive difficulties | 209 (21.9%) | 37 (19.6%) | 46 (17.4%) | 11 (13.8%) | 0.16 |
| 5. Better diagnostic and treatment strategies | 276 (28.9%) | 64 (33.9%) | 68 (25.7%) | 26 (32.5%) | 0.26 |
| 6. New/alternative treatment/s | 290 (30.4%) | 53 (28.0%) | 65 (24.5%) | 39 (48.8%) | **< .001** |
| 7. Improved phlebotomies | 105 (11.0%) | 14 (7.4%) | 29 (10.9%) | 9 (11.3%) | 0.52 |
| 8. New HC-related genes | 98 (10.3%) | 19 (10.1%) | 21 (7.9%) | 5 (6.3%) | 0.49 |
| 9. Equal access to genetic diagnosis | 89 (9.0%) | 16 (8.5%) | 16 (6%) | 17 (21.3%) | **< .001** |
| 10. Blood donation after phlebotomy | 170 (17.8%) | 18 (9.5%) | 61 (23%) | 23 (28.7%) | **< .001** |
| 11. HC epidemiology | 57 (6.0%) | 15 (7.9%) | 17 (6.4%) | 7 (8.8%) | 0.63 |
| 12. Transferrin saturation test implementation | 92 (9.6%) | 13 (6.9%) | 16 (6.0%) | 4 (5.0%) | 0.13 |
| 13. HC awareness in the general population | 137 (14.4%) | 56 (29.6%) | 72 (27.2%) | 23 (28.7%) | **< .001** |
| 14. HC awareness among medical doctors | 367 (38.5%) | 89 (47.1%) | 156 (58.9%) | 21 (26.3%) | **< .001** |
| 15. Other (not listed above) | 4 (2.1%) | 21 (2.2%) | 5 (1.9%) | 2 (2.5%) | 0.99 |

**Table S5. Answer distribution analysis compared by age**. The number and percentage of individuals within the same group that chose each option is given. The option “17 or younger” has been excluded as n<10 (see Materials and Methods). Significant differences in the Chi-square test are highlighted in bold.

|  | **18-29** | **30-39** | **40-49** | **50-59** | **60-69** | **70-79** | **80 or older** | **X^2^ p-value** |
| --- | --- | --- | --- | --- | --- | --- | --- | --- |
| 1. Arthropathies | 14 (53.8%) | 36 (39.6%) | 139 (53.7%) | 196 (47.2%) | 178 (41.7%) | 103 (43.6%) | 9 (24.3%) | **0.004** |
| 2. Hormonal problems | 2 (7.7%) | 16 (17.6%) | 22 (8.5%) | 34 (8.2%) | 31 (7.3%) | 24 (10.2%) | 3 (8.1%) | 0.1 |
| 3. Chronic fatigue | 8 (30.8%) | 26 (28.6%) | 85 (32.8%) | 133 (32%) | 104 (24.4%) | 48 (20.3%) | 5 (13.5%) | **0.003** |
| 4. Cognitive difficulties | 5 (19.2%) | 34 (37.4%) | 67 (25.9%) | 78 (18.8%) | 78 (18.3%) | 39 (16.5%) | 3 (8.1%) | **< .001** |
| 5. Better diagnostic and treatment strategies | 2 (7.7%) | 28 (30.8%) | 79 (30.5%) | 123 (29.6%) | 142 (33.3%) | 55 (23.3%) | 5 (13.5%) | **0.006** |
| 6. New/alternative treatment/s | 10 (38.5%) | 25 (27.5%) | 73 (28.2%) | 110 (26.5%) | 132 (30.9%) | 84 (35.6%) | 13 (35.1%) | 0.23 |
| 7. Improved phlebotomies | 3 (11.5%) | 4 (4.4%) | 18 (6.9%) | 39 (9.4%) | 51 (11.9%) | 37 (15.1%) | 5 (13.5%) | **0.02** |
| 8. New HC-related genes | 2 (7.7%) | 6 (6.6%) | 24 (9.3%) | 40 (9.6%) | 40 (9.4%) | 24 (10.2%) | 7 (18.9%) | 0.56 |
| 9. Equal access to genetic diagnosis | 4 (15.4%) | 10 (11%) | 17 (6.6%) | 39 (9.4%) | 42 (9.8%) | 17 (7.2%) | 5 (13.5%) | 0.43 |
| 10. Blood donation after phlebotomy | 5 (19.2%) | 18 (19.8%) | 40 (15.4%) | 89 (21.4%) | 87 (20.4%) | 31 (13.1%) | 3 (8.1%) | 0.06 |
| 11. HC epidemiology | 0 (0.0%) | 6 (6.6%) | 14 (5.4%) | 26 (6.3%) | 31 (7.3%) | 15 (6.4%) | 4 (10.8%) | 0.7 |
| 12. Transferrin saturation test implementation | 2 (7.7%) | 3 (3.3%) | 14 (5.4%) | 45 (10.8%) | 40 (9.4%) | 18 (7.6%) | 3 (8.1%) | 0.12 |
| 13. HC awareness in the general population | 4 (15.4%) | 11 (12.1%) | 45 (17.8%) | 67 (16.1%) | 93 (21.8%) | 52 (22.0%) | 15 (40.5%) | **0.003** |
| 14. HC awareness among medical doctors | 8 (30.8%) | 35 (38.5%) | 96 (37.1%) | 168 (40.5%) | 180 (42.2%) | 124 (52.5%) | 23 (62.2%) | **0.001** |
| 15. Other (not listed above) | 1 (3.8%) | 1 (1.1%) | 6 (2.3%) | 6 (1.4%) | 9 (2.1%) | 8 (3.4%) | 1 (2.7%) | 0.73 |

**Table S6. Answer distribution analysis compared by the time belonging to an HC association**. The number and percentage of individuals within the same group that chose each option is given. Significant differences in the Chi-square test are highlighted in bold.

|  | **Never** | **Less than 1 year** | **1-5 years** | **5-10 years** | **More than 10 years** | **X^2^ p-value** |
| --- | --- | --- | --- | --- | --- | --- |
| 1. Arthropathies | 0 (0.0%) | 62 (18.5%) | 466 (97.1%) | 144 (61.0%) | 4 (1.5%) | **< .001** |
| 2. Hormonal problems | 4 (2.2%) | 34 (10.1%) | 59 (12.3%) | 17 (7.2%) | 18 (6.9%) | **< .001** |
| 3. Chronic fatigue | 0 (0.0%) | 89 (26.6%) | 222 (46.3%) | 70 (29.7%) | 29 (11.2%) | **< .001** |
| 4. Cognitive difficulties | 14 (7.7%) | 84 (25.1%) | 121 (25.2%) | 47 (19.9%) | 38 (14.6%) | **< .001** |
| 5. Better diagnostic and treatment strategies | 84 (46.2%) | 121 (36.1%) | 100 (20.8%) | 53 (22.5%) | 76 (29.2%) | **< .001** |
| 6. New/alternative treatment/s | 61 (33.5%) | 113 (33.7%) | 92 (19.2%) | 76 (32.2%) | 106 (40.8%) | **< .001** |
| 7. Improved phlebotomies | 26 (14.3%) | 43 (12.8%) | 31 (6.5%) | 21 (8.9%) | 36 (13.8%) | **0.002** |
| 8. New HC-related genes | 22 (12.1%) | 38 (11.3%) | 26 (5.4%) | 15 (6.4%) | 42 (16.2%) | **< .001** |
| 9. Equal access to genetic diagnosis | 20 (11%) | 31 (9.3%) | 21 (4.4%) | 19 (8.1%) | 44 (16.9%) | **< .001** |
| 10. Blood donation after phlebotomy | 48 (26.4%) | 82 (24.5%) | 67 (14.0%) | 35 (14.8%) | 41 (15.8%) | **< .001** |
| 11. HC epidemiology | 24 (13.2%) | 20 (6.0%) | 9 (1.9%) | 10 (4.2%) | 33 (12.7%) | **< .001** |
| 12. Transferrin saturation test implementation | 24 (13.2%) | 26 (7.8%) | 20 (4.2%) | 24 (10.2%) | 31 (11.9%) | **< .001** |
| 13. HC awareness in the general population | 73 (40.1%) | 65 (19.4%) | 29 (6.0%) | 44 (18.6%) | 78 (30.0%) | **< .001** |
| 14. HC awareness among medical doctors | 119 (65.4%) | 151 (45.1%) | 141 (29.4%) | 99 (41.9%) | 125 (48.1%) | **< .001** |
| 15. Other (not listed above) | 4 (2.2%) | 7 (2.1%) | 4 (0.8%) | 3 (1.3%) | 14 (5.4%) | **0.001** |
